# Application of Deep Learning Algorithm–Based Image Reconstruction in Improving Abdominal CT Image Quality in Adrenal and Renal Lesions Detection

**DOI:** 10.1155/rrp/2214739

**Published:** 2026-08-30

**Authors:** Jingyi Tian, Hui Yao, Cong Cao, Peng Zhang, Sijia Wang, Mingyue Kang, Huafeng Cui, Su Wu

**Affiliations:** ^1^ Department of Radiology, Beijing Water Conservancy Hospital, Beijing, China; ^2^ Philips CT Clinical Science Global, Philips Health Technology, Beijing, China

**Keywords:** computed tomograph, deep learning algorithm, image quality, precise image reconstruction algorithm, renal cyst

## Abstract

**Objective:**

To evaluate the performance of the Deep Learning reconstruction algorithm Precise Image (PI) in improving abdominal computed tomography (CT) image quality and its diagnostic efficacy for adrenal and renal lesions.

**Methods:**

A total of 191 patients who underwent abdominal CT scan were retrospectively enrolled. All image datasets were reconstructed into four sequences with a slice thickness of 1 mm: Filtered back projection (FBP), Iterative Reconstruction (iDose), PI Standard, and PI Smooth. We measured CT attenuation values and the standard deviation (SD) of adrenal glands, renal parenchyma, and intra‐abdominal fat for each reconstruction group. The contrast‐to‐noise ratio (CNR) and signal‐to‐noise ratio (SNR) of adrenal and renal parenchyma were subsequently calculated. Two radiologists independently performed subjective assessments for overall image quality and diagnostic confidence for adrenal nodules and renal cysts using the Likert 5 and 4 scales, respectively. Inter‐reader agreement was quantified via the Kappa test. Intergroup comparisons were conducted using the Kruskal–Wallis test with Bonferroni post hoc correction. A priori power analysis was performed to verify the adequacy of the sample size.

**Results:**

Statistically significant intergroup differences were identified for all subjective evaluation metrics (*p* < 0.05). The image quality score increases sequentially from FBP to PI Smooth group, and PI Smooth group has the highest subjective score. There were significant differences in diagnostic confidence scores among all groups (*p* < 0.05). PI Smooth has the highest score. There were significant differences in CT values, SD values, SNR, and CNR of the adrenal glands and renal parenchyma among the four groups of reconstructed images (*p* < 0.05). SD values declined progressively from FBP to iDose and further to PI Smooth, while SNR and CNR increased in the same order. Post hoc power analysis demonstrated statistical power > 0.99 for all primary clinical endpoints, confirming robust and reproducible statistical findings.

**Conclusion:**

Compared with FBP and iDose, the PI reconstruction algorithm substantially improves abdominal CT image quality. PI Smooth delivers the highest diagnostic confidence for small lesions and renal cysts, providing robust quantitative and qualitative support for precise clinical evaluation.

**Keypoints**
1. This study investigates the clinical diagnostic applicability of the deep learning–based Precise Image reconstruction algorithm.
2. Precise Image is trained to reproduce FBP noise textures while significantly reducing image noise.
3. Compared with FBP and iDose reconstruction algorithms, the PI algorithm significantly improved the image quality and the lesion boundary, which further improved the diagnosis confidence.

## 1. Introduction

Filtered back projection (FBP) is the traditional algorithm for CT image reconstruction. Projection data are convolved with a high‐pass filter to compensate for blurring during the CT acquisition process and then back‐projected to image data. However, FBP is based on several ideal assumptions, such as a point‐like X‐ray source, pencil‐beam X‐ray geometry, detector element center points, and noise‐free projection data [1]. Therefore, FBP ignores the image noise caused by variations in Poisson photon count statistics, leading to increased image noise and artifacts when the radiation dose is reduced. To obtain high‐quality images, patients need to receive higher radiation doses [2, 3]. Currently, the commonly used image reconstruction methods in clinical practice are iterative reconstruction (IR) algorithms, such as Adaptive Statistical Iterative Reconstruction (ASiR), iDose4, Iterative model reconstruction (IMR), etc. These methods can reduce CT image noise, thereby improving image quality. However, as the number of iterations increases, the reconstructed images may change the image texture, produce edge blurring artifacts, and exhibit unnatural appearances such as excessive smoothness and a plastic‐like look, which can lead to a decrease in lesion detection rates and also reduce the reconstruction speed [4–6].

In recent years, with the significant improvement of computer computing power and the development of complex algorithms, deep learning reconstruction (DLR) algorithms can reduce image noise without changing the image texture, improve spatial resolution, enhance image quality, and further reduce radiation dose [7–9]. The Precise Image (PI) reconstruction algorithm is a novel Philips approach that uses AI for images with an appearance that more closely resembles that of typical FBP while retaining the noise‐reduction capabilities of advanced IR methods. This provides high‐quality images with a familiar appearance and at a low dose [10]. PI reconstruction is trained to quickly yield low‐noise images and substantially improved low‐contrast imaging. This study mainly assesses the image quality of the PI reconstruction algorithm in plain CT scans of the abdominal and pelvic regions; the evaluation of the PI algorithm’s ability to detect low‐contrast lesions was conducted by examining adrenal nodules and renal cysts.

## 2. Materials and Methods

### 2.1. Data Inclusion

The study was conducted in accordance with the Declaration of Helsinki (as revised in 2013). The Institutional Ethics Committee of Beijing Water Conservancy Hospital approved this study, and individual informed for this retrospective analysis was waived. This study was approved by the institutional review board. Due to the retrospective character of the data acquisition and analysis and emphasis on patient safety and quality control as well, written informed consent was waived.

**FIGURE 1:**
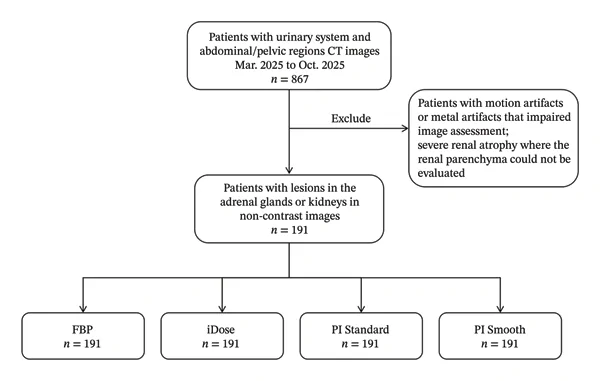
Flowchart of the study inclusion and exclusion process.

Image data of patients who underwent plain CT scans of the urinary system and abdominal/pelvic regions in our hospital from March 2025 to October 2025 (*n* = 867) were retrospectively collected. Inclusion criteria: (1) greater than 18‐year‐old adults; (2) patients with lesions in the adrenal glands or kidneys in noncontrast images; (3) patients had not received abdominal radiotherapy, chemotherapy or surgical treatment. Exclusion criteria: (1) patients with motion artifacts or metal artifacts that impaired image assessment; (2) severe renal atrophy where the renal parenchyma could not be evaluated. A total of 191 patients were enrolled, with 4 reconstruction sequences each, and a total of 764 image data (Figure 1).

### 2.2. CT Scanning Protocol

All image data were derived from a single CT device (Philips Incisive CT, Best, the Netherlands). Non‐contrast‐enhanced CT sequences were acquired or analyzed for the enrolled patients. The two main reasons for excluding the enhanced CT images are as follows: (1) all patients came for screening purposes due to abdominal pain, and noncontrast CT is the preferred screening method for patients with suspected renal lesions; (2) reduce confounding variables in image quality and feature analysis. The main scan parameters were as follows: fixed tube voltage 120 kV and automatic tube current modulation scanning, the average tube current is 144.48 ± 29.12 mAs; pitch, 0.99; rotation time, 0.5 s; detector collimation, 64 × 0.625; slice thickness, 1 mm. All images were reconstructed with FBP, iDose, PI sequences (PI Standard and PI Smooth). Window width and window level were preset at 300/70HU, then the images were recoded, randomly sorted, and provided for reading.

### 2.3. Image Evaluation

Subjective Image Assessment (SIA): Two radiologists (R1 and R2, with 8 years and 10 years of imaging diagnosis experience, respectively) independently reviewed the image data using a double‐blind method to detect lesions in the adrenal glands or kidneys, and simultaneously evaluated the image quality and diagnostic confidence. The detection of lesions was then reviewed by a deputy chief physician with 15 years of diagnostic experience. Image quality was rated on a five‐point scale as follows: 1 = high image noise, unclear lesion display, completely unable to meet clinical diagnosis requirements; 2 = high image noise, blurred lesion display, poor contrast of lesion edges, unable to fully meet clinical diagnosis requirements; 3 = relatively high image noise, clear lesion display, acceptable contrast of edges, able to meet diagnostic needs; 4 = low image noise, clear lesion display, relatively sharp edges, able to meet diagnostic needs; 5 = very low image noise, clear lesion display, clear contrast, fully meeting clinical diagnosis requirements. Diagnostic confidence was assessed on a four‐point scale: 4 = highly confident, 3 = moderately confident, 2 = probable diagnosis, 1 = uncertain, unable to diagnose.

Objective Image Assessment: To ensure the reproducibility of the results, we provided unified training on the localization of the target area to two physicians before the start of the study. Four were laid out side by side in one screen. The physician localizes the images to the same anatomical position (the renal parenchyma at the level of the second lumbar vertebra and the junction of the adrenal gland), draws the region of interest (ROI) on the FBP image, and copies and pastes each ROI to the same position on the same layer images of the iDose and PI sequences. The area of each ROI is approximately 2–3 mm^2^. The average CT value and its standard deviation (SD) value were recorded. Take the SD value of the abdominal fat in the same layer as the background noise (BN), and measure the CT values and SD values of the adrenal gland and renal parenchyma with the same‐sized ROI. Calculate the signal‐to‐noise ratio (SNR) and contrast‐to‐noise ratio (CNR) of the adrenal and renal ROI in the four groups of images and take the average of the subjective scores of the two physicians as the final score.

(1) SNRAdrenal=CTAdrenalSDFat,CNRAdrenal=CTAdrenal−CTFatBN,SNRKidney=CTKidneySDFat,CNRKidney=CTKidney−CTFatBN.

Primary endpoint: Comprehensive SIA and diagnostic confidence score (DCS) for adrenal nodules and renal cysts on abdominal noncontrast CT, aiming to compare the performance of four reconstruction algorithms (FBP, iDose, PI Standard, PI Smooth). Secondary endpoints: Objective quantitative image quality metrics (SD, SNR, CNR of adrenal gland, renal parenchyma, and abdominal fat), inter‐reader consistency, statistical power analysis, and clinical applicability analysis of PI DLR.

### 2.4. Radiation Dose

The CT dose index of volume (CTDLvol) and dose length product (DLP) generated automatically after scanning were recorded. The effective dose (ED) is calculated as follows: ED = DLP × *k*, where conversion factor *k* = 0.015 mSv/ (mGy·cm) [11].

### 2.5. Statistical Methods

Statistical analysis software SPSS 2.0 and Python 3 (scipy.stats) were used for data analysis. The Kolmogorov–Smirnov method was used to assess the normality of continuous quantitative variable. Quantitative variables are presented as the mean (SD) for normalized data and as the median (first quartile, third quartile) for non‐normalized data. The Kruskal–Wallis test was used for multigroup comparison, and the Wilcoxon rank sum test was used for comparison between groups. The Bonferroni correction method was used to adjust the significance level (*α* = 0.05/number of comparisons) when comparing diagnostic performance and objective metrics among the four reconstruction groups (FBP, iDose, PI Standard, PI Smooth). Effect sizes were systematically calculated and reported alongside statistical significance: for Kruskal–Wallis H tests, overall effect sizes were evaluated using Kendall’s W/eta‐squared (*η* ^2^_*H* = (*H* ‐ *k* + 1)/(*N* ‐ *k*)); for pairwise comparisons, rank‐biserial correlation effect sizes were computed as *r* = $\left|Z\right|/\sqrt{N}$ (where *r* ≥ 0.1, ≥ 0.3, and ≥ 0.5 represent small, medium, and large effect sizes, respectively). Prior to study initiation, an a priori power analysis was performed using G^∗^Power software (version 3.1.9.7) to determine required sample size (*α* = 0.05, 1 ‐ *β* = 0.80, effect size *f* = 0.25, minimum required *N* = 180). Post hoc–achieved power analysis was subsequently conducted based on the final sample size (*n* = 191) and observed data to confirm statistical validity. Inter‐reader agreement for subjective SIA and DCS ratings was quantified using the weighted Kappa test. A two‐tailed *p* value < 0.05 was defined as statistically significant.

## 3. Results

### 3.1. Clinical Characteristics

**FIGURE 2:**
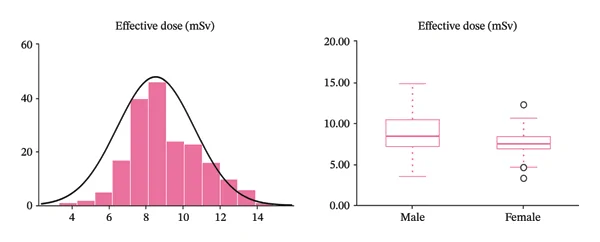
Radiation dose characteristics. The left figure shows the histogram of effective dose distribution; the right figure presents the comparison of effective doses for different genders.

A total of 191 patients (146 males, 45 females, mean age 59.65 ± 15.51 years) were enrolled. The mean tube current, CTDLvol, DLP and ED of abdominal pelvic CT of 191 patients were (144.48 ± 29.12) mAs, (11.76 ± 2.36) mGy, (565.09 ± 141.79) mGy cm, and (8.48 ± 2.13) mSv; the ED for males was slightly higher than that for females (about 14.76%–17.33%) (Table 1 and Figure 2).

**TABLE 1:** Patient demographics and radiation dose parameters.

Characteristic	Value (*n* = 191)
Age (years)	59.65 ± 15.51
Sex	
Male	146
Female	45
Tube voltage (kVp)	120
Tube current–time product (mAs)	144.56 ± 29.02
CTDIvol (mGy)	11.77 ± 2.35
DLP (mGy·cm)	566.67 ± 138.98

### 3.2. SIA

**FIGURE 3:**
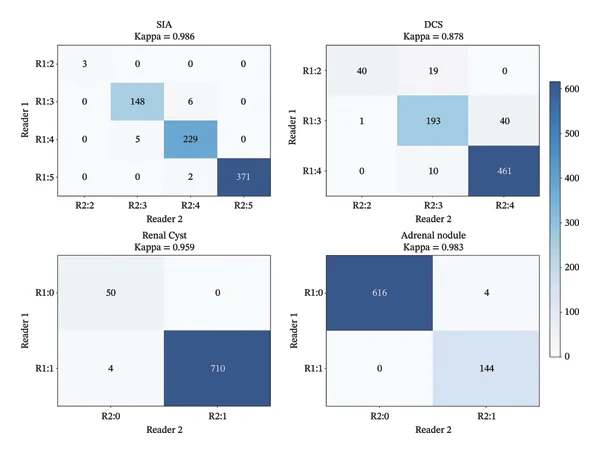
The confusion matrix of the subjective evaluation results of the four groups of reconstructed images by two physicians.

The two physicians’ evaluations of the image quality of the four groups of reconstructed images (FBP, iDose, PI Standard, and PI Smooth) showed a high degree of consistency, with a Kappa value of 0.99 (95% CI: 0.98–0.99) (Figure 3). The image quality scores increased sequentially from FBP to PI Smooth, and the subjective score of the PI Smooth group was the best (overall *η* ^2^_*H* = 0.926 for SIA, *η* ^2^_*H* = 0.465 for DCS, both *p* < 0.001). After multiple comparisons, it was found that all pairwise comparisons between FBP, iDose, and PI Standard remain statistically significant with large effect sizes (*p* < 0.0083, *r* = 0.349–0.864), while the comparison between PI Standard and PI Smooth is not significant with negligible effect size (*p* > 0.0083, *r* = 0.019–0.032) for SIA and DCS, consistent with their similar clinical performance (Table 2).

**TABLE 2:** Subjective assessment scores and objective image quality metrics across four CT reconstruction groups.

Variable	FBP (*n* = 191)	iDose (*n* = 191)	PI standard (*n* = 191)	PI smooth (*n* = 191)	H value (*p* value)
SIA Median (Q1, Q3)	3.00 (3.00, 3.00)	4.00 (4.00, 4.00)	5.00 (5.00, 5.00)	5.00 (5.00, 5.00)	*H* = 706.74 (*p* < 0.001)
DCS Median (Q1, Q3)	3.00 (3.00, 3.00)	3.50 (3.00, 4.00)	4.00 (4.00, 4.00)	4.00 (4.00, 4.00)	*H* = 356.53 (*p* < 0.001)

**Post-hoc pairwise comparisons (Mann–Whitney U, Bonferroni-corrected; *α* = 0.05/6 = 0.0083)**					
**Pairwise comparison**	**SIA**	**DCS**	**SD (Kidney)**	**SD (Adrenal)**	**SD (Fat)**

FBP vs iDose	*U* = 4170.5, *p* < 0.001^∗^, *r* = 0.667	*U* = 10885.5, *p* < 0.001^∗^, *r* = 0.349	*U* = 31663.5, *p* < 0.001^∗^, *r* = 0.636	*U* = 28254.0, *p* < 0.001^∗^, *r* = 0.472	*U* = 31048.0, *p* < 0.001^∗^, *r* = 0.607
FBP vs PI Standard	*U* = 148.0, *p* < 0.001^∗^, *r* = 0.858	*U* = 3967.5, *p* < 0.001^∗^, *r* = 0.677	*U* = 36385.5, *p* < 0.001^∗^, *r* = 0.860	*U* = 34544.5, *p* < 0.001^∗^, *r* = 0.772	*U* = 36175.0, *p* < 0.001^∗^, *r* = 0.850
FBP vs PI Smooth	*U* = 18.5, *p* < 0.001^∗^, *r* = 0.864	*U* = 3659.5, *p* < 0.001^∗^, *r* = 0.691	*U* = 36481.0, *p* < 0.001^∗^, *r* = 0.865	*U* = 36111.0, *p* < 0.001^∗^, *r* = 0.847	*U* = 36480.0, *p* < 0.001^∗^, *r* = 0.865
iDose vs PI Standard	*U* = 732.0, *p* < 0.001^∗^, *r* = 0.830	*U* = 9370.0, *p* < 0.001^∗^, *r* = 0.421	*U* = 34342.5, *p* < 0.001^∗^, *r* = 0.763	*U* = 29173.0, *p* < 0.001^∗^, *r* = 0.516	*U* = 32834.5, *p* < 0.001^∗^, *r* = 0.692
iDose vs PI Smooth	*U* = 91.5, *p* < 0.001^∗^, *r* = 0.861	*U* = 8966.0, *p* < 0.001^∗^, *r* = 0.440	*U* = 36464.0, *p* < 0.001^∗^, *r* = 0.864	*U* = 34569.5, *p* < 0.001^∗^, *r* = 0.773	*U* = 36357.0, *p* < 0.001^∗^, *r* = 0.859
PI Standard vs PI Smooth	*U* = 17568.5, *p* = 0.190 (NS), *r* = 0.032	*U* = 17842.5, *p* > 0.999 (NS), *r* = 0.019	*U* = 35151.0, *p* < 0.001^∗^, *r* = 0.802	*U* = 29622.0, *p* < 0.001^∗^, *r* = 0.537	*U* = 33660.0, *p* < 0.001^∗^, *r* = 0.731

^∗^Significant after Bonferroni correction.

**FIGURE 4:**
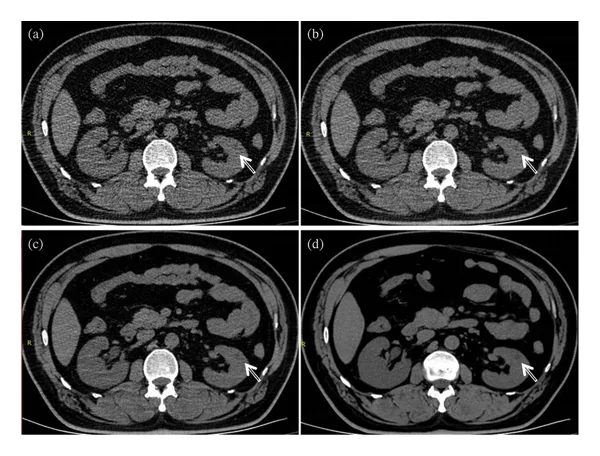
Comparative analysis of image quality differences among four reconstruction algorithms (FBP, iDose, PI Standard, and PI Smooth) in abdominal CT imaging. The images were derived from an 89‐year‐old male who underwent plain abdominal CT scanning, with corresponding reconstructed images using FBP (a), iDose (b), PI Standard (c), and PI Smooth (d); the subjective image quality scores of these images were 2, 3, 4, and 5, respectively. On the plain CT images, a cystic low‐density lesion is visible in the renal parenchyma of the left kidney.

**FIGURE 5:**
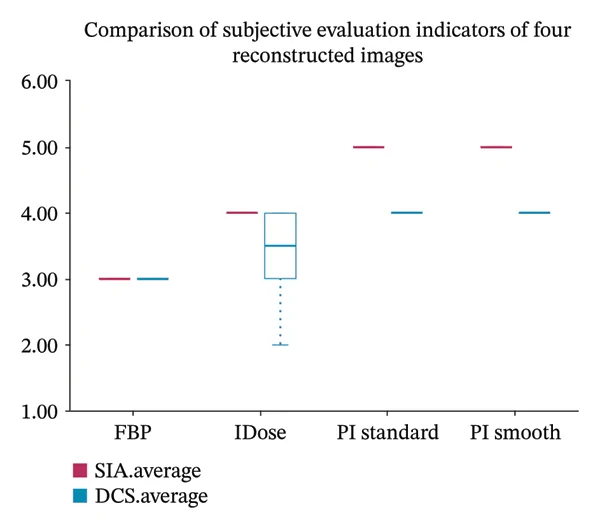
Distribution of scores by group (FBP, iDose, PI Standard, and PI Smooth). The boxes represent the interquartile range (IQR), with the median score indicated by the horizontal line within each box. Whiskers extend to the smallest and largest values within 1.5 times the IQR. Diagnostic confidence score (DCS) and subjective image assessment (SIA) of PI group is highest among four groups.

The diagnostic confidence in the detection of the overall lesion was consistent among different readers; the inter‐reader consistency coefficient was 0.88 (95% confidence interval: 0.85–0.91), indicating a relatively high level of consistency. There was a statistically significant difference in DCSs among the FBP, iDose, PI Standard, and PI Smooth (*p* < 0.05), while there was no statistically significant difference between the PI Standard and PI Smooth groups (*p* > 0.05), all illustrated in Figures 3, 4, and 5.

### 3.3. Objective Image Assessment

The intraclass correlation coefficient (ICC) was calculated to evaluate the intergroup reliability of each quantitative parameter. The results showed that the two physicians had high consistency in their evaluations of the image quality of the four groups of reconstructed images (FBP, iDose, PI Standard, and PI Smooth) (Table 3).

**TABLE 3:** Comparison of objective assessment of four reconstructed images.

	FBP	iDose	PI standard	PI smooth	*p* value
CT value					
Kidney (ICC)	0.869	0.897	0.928	0.946	
R1	32.00 (30.0,34.0)	32.00 (30.0,34.0)	31.00 (29.0,33.0)	31.00 (28.0,33.0)	< 0.001
R2	33.00 (31.0,34.0)	32.00 (31.0,34.0)	32.00 (30.0,33.0)	31.00 (29.0,33.0)	< 0.001
avg	32.50 (31.0,34.0)	32.00 (30.5,33.5)	32.00 (30.0,33.0)	31.00 (29.0,33.0)	< 0.001
Adrenal gland (ICC)	0.855	0.900	0.931	0.944	
R1	29.00 (25.0,33.0)	27.00 (23.0,31.0)	29.00 (24.0,33.0)	28.00 (24.0,32.0)	0.047
R2	30.00 (28.0,31.0)	28.00 (26.0,30.0)	29.00 (26.0,31.0)	28.00 (26.0,31.0)	< 0.001
avg	29.50 (27.0,32.0)	28.00 (25.0,30.5)	28.50 (25.5,32.0)	28.00 (25.5,31.5)	0.001
Abdominal fat (ICC)	0.961	0.965	0.971	0.974	
R1	−104.00 (−108.0,‐99.0)	−104.00 (−108.0,‐99.0)	−105.00 (−109.0,‐99.0)	−105.00 (−109.0,‐99.0)	0.877
R2	−104.00 (−107.0,‐100.0)	−105.00 (−108.0,‐100.0)	−105.00 (−108.0,‐100.0)	−105.00 (−108.0,‐100.0)	0.880
avg	−104.00 (−108.0,‐100.0)	−104.50 (−108.0,‐100.0)	−104.50 (−108.5,‐100.0)	−104.50 (−108.5,‐100.0)	0.869
SD value					
Kidney (ICC)	0.923	0.919	0.915	0.886	
R1	27.20 (23.9,29.9)	21.00 (18.3,23.1)	14.60 (12.9,16.2)	9.70 (8.6,10.9)	< 0.001
R2	28.00 (24.7,31.5)	21.50 (19.2,24.4)	15.10 (13.5,16.9)	10.10 (9.0,11.3)	< 0.001
avg	27.50 (24.8,30.4)	21.40 (19.1,23.5)	15.05 (13.3,16.8)	9.90 (8.8,11.1)	< 0.001
Adrenal gland (ICC)	0.959	0.953	0.948	0.932	
R1	29.70 (25.1,34.0)	23.20 (19.7,26.9)	17.80 (15.3,20.2)	13.30 (10.6,15.5)	< 0.001
R2	28.90 (24.6,34.1)	22.40 (19.1,27.0)	17.20 (14.7,20.2)	12.60 (10.5,14.7)	< 0.001
avg	29.30 (24.9,33.2)	23.05 (19.7,26.6)	17.30 (15.2,20.2)	12.90 (10.8,15.1)	< 0.001
Abdominal fat (ICC)	0.941	0.934	0.918	0.885	
R1	26.30 (23.3,30.5)	20.40 (18.0,23.7)	15.00 (13.2,16.7)	10.50 (9.2,12.1)	< 0.001
R2	27.10 (24.6,30.7)	21.10 (19.2,24.0)	15.20 (13.9,17.3)	10.60 (9.6,12.2)	< 0.001
avg	27.20 (24.6,30.4)	20.90 (18.9,23.5)	15.20 (13.8,17.0)	10.60 (9.6,12.0)	< 0.001
SNR					
Kidney (ICC)	0.906	0.911	0.914	0.907	
R1	1.19 (1.1,1.4)	1.51 (1.4,1.8)	2.10 (1.9,2.5)	3.16 (2.8,3.6)	< 0.001
R2	1.16 (1.0,1.3)	1.49 (1.3,1.7)	2.06 (1.8,2.4)	3.09 (2.7,3.5)	< 0.001
avg	1.19 (1.1,1.4)	1.51 (1.4,1.8)	2.10 (1.9,2.5)	3.16 (2.8,3.6)	< 0.001
Adrenal gland (ICC)	0.900	0.906	0.908	0.903	
R1	0.99 (0.8,1.2)	1.17 (0.9,1.5)	1.60 (1.3,2.0)	2.11 (1.7,2.8)	< 0.001
R2	1.01 (0.8,1.2)	1.21 (1.0,1.5)	1.65 (1.4,2.0)	2.24 (1.8,2.8)	< 0.001
avg	0.99 (0.8,1.2)	1.17 (0.9,1.5)	1.60 (1.3,2.0)	2.11 (1.7,2.8)	< 0.001
CNR					
Kidney (ICC)	0.953	0.950	0.942	0.919	
R1	5.14 (4.6,5.7)	6.65 (5.9,7.4)	9.26 (8.2,10.3)	13.43 (11.8,15.0)	< 0.001
R2	4.96 (4.4,5.6)	6.40 (5.6,7.3)	8.81 (7.9,10.0)	12.85 (11.5,15.0)	< 0.001
avg	5.14 (4.6,5.7)	6.65 (5.9,7.4)	9.26 (8.2,10.3)	13.43 (11.8,15.0)	< 0.001
Adrenal gland (ICC)	0.959	0.958	0.950	0.934	
R1	4.76 (4.1,5.4)	6.01 (5.2,6.8)	7.95 (6.9,9.3)	10.89 (9.6,12.7)	< 0.001
R2	4.69 (4.1,5.4)	5.92 (5.1,6.8)	7.91 (7.1,9.3)	11.11 (9.8,12.8)	< 0.001
avg	4.74 (4.1,5.3)	6.01 (5.2,6.8)	7.95 (6.9,9.3)	10.89 (9.6,12.7)	< 0.001

### 3.4. Subgroup Analysis of the Lesion Site

The kidney noise of PI Smooth is only 9.90, which is 63.7% lower than that of FBP, 53.0% lower than that of iDose, and 33.2% lower than that of PI Standard. Its noise control ability is the best among the four technologies. The SNR and CNR of the kidneys in the PI Smooth method reached 3.16 and 13.43, respectively, which were 164% and 160% higher than those of FBP, and 106% and 102% higher than those of iDose. The contrast between the renal cyst and the surrounding renal tissue was significantly improved, and the boundary of the lesion became clearer.

The adrenal noise of PI Smooth is only 12.90, which is 54.5% lower than that of FBP, 42.3% lower than that of iDose, and 24.7% lower than that of PI Standard. Its noise control ability is also the best. The adrenal SNR and CNR of PI Smooth reached 2.11 and 13.43, respectively, which were 116% and 140% higher than those of FBP, and 78% and 90% higher than those of iDose. The contrast between adrenal nodules and surrounding tissues was significantly improved, and the display effect for low‐contrast adrenal nodules was particularly remarkable.

**FIGURE 6:**
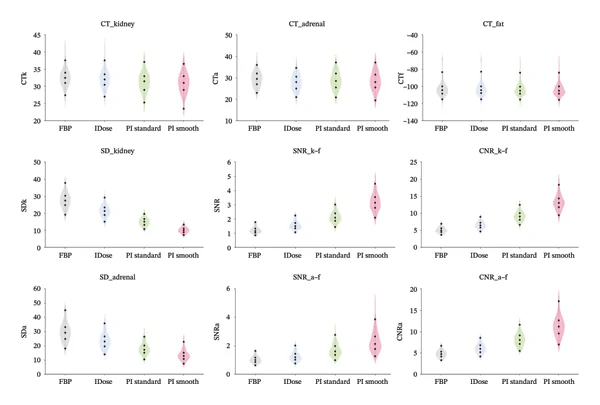
The violin plot separately presents the CT values, SD values, SNR, and CNR of the kidneys and adrenal glands. Among the four sets of reconstructed images, there were no statistically significant differences in the measured values of each tissue. The SD values of the adrenal glands and kidneys in the PI Smooth reconstructed images were the lowest, while the SNR and CNR values outperformed those of the FBP, iDose, and PI Standard reconstructed images.

For all objective metrics (SD, SNR, CNR in kidney, adrenal, and fat tissues), overall four‐group comparisons demonstrated highly significant differences with large effect sizes (*η* ^2^_*H* = 0.759–0.854, all *p* < 0.001). In post hoc pairwise comparisons, all pairwise comparisons between FBP, iDose, and PI Standard are statistically significant (*p* < 0.001 < 0.0083), with large effect sizes (*r* = 0.421–0.865), with PI algorithms showing substantially lower noise and higher SNR/CNR than FBP and iDose. Even PI Standard vs PI Smooth show significant differences for all objective metrics (*p* < 0.001 < 0.0083, *r* = 0.463–0.802). This indicates the two PI algorithms have measurable differences in physical image quality characteristics (Table 4, Table 5, and Figure 6).

**TABLE 4:** Subjective assessment scores and objective image quality metrics across four CT reconstruction groups.

Pairwise comparison	SNR (kidney)	SNR (adrenal)	CNR (kidney)	CNR (adrenal)	H (*P* value)
FBP vs iDose	*U* = 6493.5, *p* < 0.001^∗^, *r* = 0.555	*U* = 10934.0, *p* < 0.001^∗^, *r* = 0.347	*U* = 4869.0, *p* < 0.001^∗^, *r* = 0.633	*U* = 6913.0, *p* < 0.001^∗^, *r* = 0.535	*H* = 620.12 (*p* < 0.001)
FBP vs PI Standard	*U* = 574.0, *p* < 0.001^∗^, *r* = 0.838	*U* = 3043.0, *p* < 0.001^∗^, *r* = 0.721	*U* = 148.0, *p* < 0.001^∗^, *r* = 0.858	*U* = 651.0,*p* < 0.001^∗^, *r* = 0.834	*H* = 652.32 (*p* < 0.001)
FBP vs PI Smooth	*U* = 0.0, *p* < 0.001^∗^, *r* = 0.866	*U* = 535.0, *p* < 0.001^∗^, *r* = 0.840	*U* = 0.0, *p* < 0.001^∗^, *r* = 0.866	*U* = 14.0, *p* < 0.001^∗^, *r* = 0.865	*H* = 644.41 (*p* < 0.001)
iDose vs PI Standard	*U* = 4035.0, *p* < 0.001^∗^, *r* = 0.673	*U* = 7467.0, *p* < 0.001^∗^, *r* = 0.509	*U* = 2519.0, *p* < 0.001^∗^, *r* = 0.745	*U* = 4697.0, *p* < 0.001^∗^, *r* = 0.642	*H* = 608.11 (*p* < 0.001)
iDose vs PI Smooth	*U* = 149.0, *p* < 0.001^∗^, *r* = 0.858	*U* = 2162.0, *p* < 0.001^∗^, *r* = 0.763	*U* = 26.0, *p* < 0.001^∗^, *r* = 0.864	*U* = 467.0, *p* < 0.001^∗^, *r* = 0.843	*H* = 579.53 (*p* < 0.001)
PI Standard vs PI Smooth	*U* = 2611.0, *p* < 0.001^∗^, *r* = 0.741	*U* = 8446.0, *p* < 0.001^∗^, *r* = 0.463	*U* = 1779.0, *p* < 0.001^∗^, *r* = 0.780	*U* = 4617.0, *p* < 0.001^∗^, *r* = 0.646<	*H* = 447.14 (*p* < 0.001)

*Note:* SD, standard deviation (noise).

Abbreviations: CNR, contrast‐to‐noise ratio; DCS, diagnostic confidence score; HU, Hounsfield unit; SIA, subjective image assessment; SNR, signal‐to‐noise ratio.

^∗^Significant after Bonferroni correction.

**TABLE 5:** A priori power analysis (G^∗^Power 3.1.9.7) for the primary endpoint (adrenal nodule and renal cyst diagnostic confidence).

Primary endpoint (metric)	*η* ^2^	Cohen’s w	Effect size	Power (achieved, *n* = 191)	Conclusion
SIA	0.9263	3.544	Very large	> 0.99	Sufficient power confirmed
DCS	0.4673	0.937	Large	> 0.99	Sufficient power confirmed
SD (kidney, noise)	0.8549	2.428	Very large	> 0.99	Sufficient power confirmed
SNR (kidney)	0.7970	1.981	Very large	> 0.99	Sufficient power confirmed
CNR (kidney)	0.8446	2.331	Very large	> 0.99	Sufficient power confirmed
SD (adrenal)	0.6451	1.347	Very large	> 0.99	Sufficient power confirmed
SNR (adrenal)	0.5860	1.189	Large	> 0.99	Sufficient power confirmed
CNR (adrenal)	0.7595	1.777	Very large	> 0.99	Sufficient power confirmed

### 3.5. Power Analysis and Sample Size Validation

An a priori sample size estimation indicated that a minimum of 180 patients per group would provide 80% power at *α* = 0.05. In our study, a final cohort of *n* = 191 patients per group (764 dataset reconstructions in total) was evaluated. Subsequent post hoc–achieved power calculations based on the observed effect sizes confirmed that all primary endpoints (SIA, DCS, SD, SNR, and CNR) achieved statistical power > 0.99 (Table 6), far exceeding the conventional minimum power threshold of 0.80 for clinical research. This confirms that the study sample size is more than sufficient to detect true differences between the reconstruction groups. The statistically significant findings in the pairwise comparisons are highly reliable and replicable.

**TABLE 6:** Comparative overview of manufacturer‐specific DLR algorithms.

Feature/manufacturer	GE Healthcare (DLIR/TrueFidelity)	Siemens Healthineers (ADMIRE)	Philips (precise image/AI‐DLR)	Canon Medical Systems (AiCE)
Core technology	Deep learning image reconstruction (DLIR) [12]	Model iterative reconstruction (MDIR) [13]	Artificial intelligence deep learning reconstruction (AI‐DLR) [14]	Advanced intelligent Clear‐IQ Engine (AiCE) [15]

Key advantage	Superior noise reduction and image quality at low doses, preserving image texture and detail [12]	Improved low‐contrast detectability and noise reduction, especially for brain imaging and dual‐energy applications [16]	Significant noise reduction and improved task‐based image quality, retains the authentic texture characteristic of traditional FBP [14]	Excellent noise suppression and clarity, even at very low radiation exposures; available in multiple strength levels [15, 17]

Comparison to IR	Outperforms ASiR‐V in noise reduction and image quality metrics (e.g., CNR, SNR) [18, 19]	Shows improved low‐contrast detectability over FBP and ADMIRE [16]	Demonstrates superior image quality compared to hybrid IR (iDose4) in abdominal CT [14]	Better noise reduction and image quality than AIDR 3D [15]

Dose reduction	Significant dose reduction potential (total achievable: 60%–75%) [20]	Facilitates low‐dose protocols with maintained diagnostic accuracy, particularly in challenging imaging scenarios like obese patients or metal artifact reduction [21]	Offers opportunities for substantial dose reduction in abdominal CT protocols [14]	Allows for considerable dose reduction, making sub‐millisievert imaging feasible [15]

Clinical areas	Abdominal CT [19, 22], single and dual‐energy CT [23], liver tumors [24], urinary tract stones [23]	Brain CT [25], Abdominal CT [26], Coronary artery calcium (CAC) [27]	Brain CT [28], Abdominal CT [14, 22], Lung CT, Abdominal lesion detection [29]	Cardiac CT [15, 30], brain protocols [17, 31]

	DLIR‐L, DLIR‐M, DLIR‐H (low, medium, high)	ADMIRE Strength 3, Strength 4, Strength 5	Smoother, Smooth, Standard	Mild, Standard, Strong

## 4. Discussion

Optimization of CT image quality constitutes a central translational research priority for advancing clinical CT technology. DLR overcomes core limitations inherent to traditional IR: repeated IR iterations degrade anatomical edge sharpness, introduce unnatural plastic‐like tissue texture, and slow reconstruction throughput, Philips’ proprietary PI DLR algorithm is trained on paired matched high‐dose/low‐dose CT volumetric datasets, embedding simulated physical noise priors to emulate high‐dose scanning performance without elevating patient radiation exposure. PI produces thin‐slice CT volumes with suppressed BN, superior low‐contrast lesion detectability, and native FBP‐like organic tissue texture, balancing diagnostic interpretability and radiation protection [10, 14]. Based on the available information in the market, Table 6 briefly summarizes the differences between PI and other major DLR methods.

From the analysis of objective evaluation indicators, as the denoising intensity increases, the SD values of the adrenal gland and renal parenchyma gradually decrease, and SNR and CNR significantly improve. PI Smooth outperformed FBP and iDose across every subjective and objective imaging endpoint. Relative to conventional FBP reconstruction, PI Smooth reduced renal tissue noise by 63.7% and adrenal gland noise by 54.5%, with SNR and CNR improvements exceeding 120% for both anatomical sites. In routine clinical practice, subcentimeter (< 10 mm) renal microcysts are frequently missed on FBP and standard iterative CT reconstructions. PI Smooth drastically amplifies cyst‐parenchymal contrast via elevated CNR values, minimizing false‐negative lesion interpretations and eliminating unnecessary follow‐up contrast‐enhanced CT examinations. The magnitude of PI‐mediated CNR improvement was greater for adrenal nodule subgroups than renal cyst cohorts, indicating that PI delivers more pronounced optimization for intrinsically low‐contrast lesions and holds particular clinical value for adrenal nodule characterization. PI Smooth generated the sharpest lesion borders for renal cysts and adrenal nodules and achieved the highest radiologist diagnostic confidence, consistent with prior published work by Joel et al. [14, 32].

From the subjective scoring results of our study, the PI algorithms clearly delineate renal cyst and adrenal nodule boundaries and raise lesion detection rates (Figure 4). Among the different levels of PI algorithms, PI Smooth offered the sharpest small anatomical structures and lesion edges, outperforming iDose and PI Standard on abdominal low‐contrast lesion visualization (Figure 6). This also confirms that when using the phantom model to evaluate the detectability index of simulated abdominal lesions, it was found that PI reconstruction (especially at the Standard and Smooth levels) significantly improved the detectability of simulated abdominal lesions, outperforming FBP and iDose [14, 33]. In addition, PI has strong noise suppression performance. Compared with FBP, the noise reduction of PI varies from approximately 2% at the Sharper level to 80% at the Smoother level, and the noise decreases linearly with the reconstruction level (from Sharper to Smoother) [1].

In actual clinical practice, the PI algorithm will assist clinical work in the following aspects: (1) Clinical workflow integration: The PI algorithm can be embedded into existing CT postprocessing workstations, requiring no additional hardware upgrades, and can automatically process images within 10 s per case, ensuring it does not delay clinical workflow [1]. (2) Target population prioritization: Ideal for routine abdominal screening, serial renal/adrenal lesion follow‐ups, and radiation‐sensitive groups (young patients, pregnant women, patients requiring repeated CT scans), supporting low‐dose CT protocols. (3) Diagnostic benefits: Superior image clarity improves radiologists’ identification of subtle small lesions, lowering missed diagnosis rates and unnecessary follow‐up scans to optimize clinical resource allocation and patient prognosis.

There are several limitations in this study. First, all the CT scans were obtained from the same supplier platform (Philips Incisive CT) in a single center. Differences in hardware design, reconstruction algorithms, and scanning parameters among different manufacturers may lead to variations in image quality and features. Multicenter or multimanufacturer studies may provide more valuable information of DLR. Secondly, another limitation of this study is that we only evaluate the performance of PI conventional dose and did not verify the clinical efficacy of each reconstruction algorithm of PI by reducing the radiation dose. At last, the key limitations of this study lie in the lack of pathological confirmation for renal cysts and adrenal lesions, as this study relies on imaging diagnosis (the gold standard in clinical practice) rather than histopathological results. In future studies, the diagnostic value of this algorithm will be further verified by combining pathological results [34].

In summary, the PI DLR algorithm achieves meaningful, statistically significant improvements in abdominopelvic CT image quality relative to conventional FBP and iterative iDose reconstruction pipelines. The PI Smooth preset delivers maximal diagnostic confidence for small adrenal and renal lesions, providing robust quantitative and qualitative support for accurate clinical radiological evaluation.

**Nomenclature**
FBP: Filtered back projection
iDose: Iterative Reconstruction
PI: Precise Image
DLR: Deep learning reconstruction
CT: Computed tomography
SD: Standard deviation
CNR: Contrast‐to‐noise ratio
SNR: Signal‐to‐noise ratio
ROI: Region of interest
BN: Background noise
DLP: Dose length product
CTDLvol: CT dose index of volume
ED: Effective dose
SIA: Subjective image assessment
DCS: Diagnostic confidence scores

## Funding

No funding was received for this manuscript.

## Conflicts of Interest

The authors declare no conflicts of interest.

## Data Availability

The data that support the findings of this study are available upon request from the corresponding author. The data are not publicly available due to privacy or ethical restrictions.
